# A ’compensatory selection’ effect with standardized tests: Lack of correlation between test scores and success is evidence that test scores are predictive of success

**DOI:** 10.1371/journal.pone.0265459

**Published:** 2022-05-19

**Authors:** David E. Huber, Andrew L. Cohen, Adrian Staub

**Affiliations:** Department of Psychological and Brain Sciences, University of Massachusetts, Amherst, Massachusetts, United States of America; Arizona State University, UNITED STATES

## Abstract

We introduce the statistical concept of ’compensatory selection’, which arises when selecting a subset of applicants based on multiple predictors, such as when standardized test scores are used in combination with other predictors required in a school application (e.g., previous grades, references letters, and personal statements). Post-hoc analyses often fail to find a positive correlation between test scores and subsequent success, and this failure is sometimes taken as evidence against the predictive validity of the standardized test. The present analysis reveals that the *failure to find a negative correlation* indicates that the standardized test is in fact a valid predictor of success. This is due to compensation between predictors during selection: Some students are admitted despite a low test score because their application is exceptional in other respects, while other students are admitted primarily based on a high test score despite weakness in the rest of their application. This compensatory selection process introduces a negative correlation between test scores and other predictors among those admitted (a ’collider bias’ or ’Berkson’s paradox’ effect). If test scores are valid predictors of success, this negative correlation between the predictors counteracts the positive correlation between test scores and success that would have been observed if all applicants were admitted. If test scores are not predictive of success, but were nevertheless used in a compensatory selection process, there would be a spurious negative correlation between test scores and success (i.e., an admitted student with a weak application except for a high test score would be unlikely to succeed). The selection effect that is described here is fundamentally different from the well-known ’restricted range’ problem and can powerfully alter results even in situations that accept most applicants.

## Introduction

Standardized tests are designed to evaluate the academic potential and achievement of individuals compared to the population, and are often used along with other predictors such as grades, reference letters, and personal statements, to guide the selective admissions of students into degree programs. They are useful precisely because they are standardized–they provide standard scores than can help admissions decisions when reviewing applicants from a wide variety of backgrounds. For instance, admissions committee members may find it difficult to evaluate reference letters and grades provided by a lesser-known institution, but in such a case, a high standardized test score provides some reassurance. For this reason, standardized tests are used in college and university admissions around the world, including A-levels in the United Kingdom, the College Scholastic Ability Test in South Korea, the Abitur in Germany, the National College Entrance Examination in the Peoples Republic of China, the GED in Canada, the ATAR in Australia, the Vestibular in Brazil, the Baccalaureate in France, and the SAT in the United States, to name just a few.

The validity and usefulness of standardized tests has been questioned in the last decade, and many institutions of higher education are moving toward “test optional” applications. In some cases, this policy shift is based on retrospective analyses of the relationship between standardized test scores and subsequent academic success, which reveal weak or absent positive correlations, suggesting that standardized tests are poor predictors of future academic success [1–3]. However, because not every applicant is accepted, the admissions process introduces a powerful selection effect, which we term ’compensatory selection’. Below we explain the statistical concept of compensatory selection, which is fundamentally different from the well-known problem of restricted range. Using simulations of different admissions policies, we demonstrate how compensatory selection can eliminate positive correlations between test scores and success when a standardized test is, in fact, valid (i.e., it is predictive of success among the pool of applicants). Furthermore, *compensatory selection necessarily produces negative correlations between test scores and success if the standardized test is invalid*. Thus, the failure to observe a negative correlation supports the validity of the test, in a ‘compensatory selection’ environment.

### Compensation between predictors

Academic admissions involve *compensation*: A good score on one predictor may offset a poor score on another. To provide some intuition about how compensation influences the interpretation of correlational analyses, we use an analogy from the sports world: a scenario in which a factor that clearly predicts success, in general, does not predict success among those selected to participate. Consider the relationship between a player’s height and success in basketball. No one would question that height is relevant to success as a basketball player. Yet, our analysis of the 2018 National Basketball Association (NBA) season found that *height does not predict success among NBA players*. There is no significant correlation between height and an all-around measure of a player’s contribution, a statistic known as "Win Shares,” nor is there a significant correlation between height and points scored. The sample size in this analysis was fairly large (540 NBA players) and this failure to find a correlation did not reflect a restricted range; while NBA players are generally quite tall, there is considerable variability, with heights ranging from 5’ 9” to 7’ 3”. A skeptic might point out that the different positions in basketball tend to select players of different heights and this would work against such a correlation (e.g., centers tend to be taller than guards and yet guards have more opportunity to score 3-point baskets). However, a similar lack of correlation is even found within positions, i.e., guard, forward, center.

What explains this result? Have professional basketball teams been misguided in their selection of tall players? Before reaching this conclusion, note that in addition to height, success in basketball requires, among other things, good hand-eye coordination, fitness, and a strong work ethic, with these latent (unobservable) characteristics measured with observable metrics from players’ college careers, such as points per game, minutes played, field goal percentage, assists, steals, etc. To be selected for the NBA, a shorter player must compensate by excelling in most or all of these other areas. On the other hand, a very tall player might be selected for the NBA based primarily on his height. Thus, height loses its predictive power among *players admitted* to the NBA because, on average, shorter NBA players excel in these other attributes.

Similarly, an applicant to an institution of higher education with lower standardized scores who is nonetheless admitted is likely to possess compensatory qualifications, as evidenced by other measures in their application such as their grades, references letters, or personal statement. Among the pool of *admitted* students, students with lower standardized scores will tend to be exceptionally strong in other areas, and this works against observing a positive correlation between test scores and success. The example of basketball selection is used because height is obviously a factor that relates to basketball success and because height is an easily observed characteristic. In contrast, standardized tests measure latent characteristics, and the hope is that components of an application collectively capture the relevant latent characteristics that underlie academic success. Below we present computer simulations to examine whether compensatory selection can produce similar effects with latent characteristics, which necessarily involve a higher degree of uncertainty (e.g., retaking the same standardized test might yield different results) and when predictors may correlate with each other because they measure some of the same latent characteristics.

### Restricted range versus compensatory selection

When selecting a subset of individuals using a standardized measure, there are several statistical interpretation problems that can arise. For instance, in a ’regression towards the mean’ effect, selecting individuals with extreme test scores on a standardized measure can lead to interpretation problems because these same individuals will likely produce less extreme scores on a subsequent re-test, simply because of imperfect test-retest reliability [4]. In another example, selecting individuals with test scores falling within a narrow ’restricted range’ can cause a failure to find reliable correlations because a correlation is mathematically impossible if the variance of the predictor is due only to imperfect test-retest reliability, rather than from actual differences between individuals [5–7]. At first glance, it may appear that ’compensatory selection’ is the same as a restricted range effect, but this is not the case. For instance, compensatory selection can alter the results of a correlation analysis even when the selected sample covers the full range of possible test scores.

To make clear this distinction, consider the correlation coefficient in Eq 1, which compares the covariance (COV) between test scores T and subsequent academic success S, to the multiplication of the standard deviations of T (σ_T_) and S (σ_S_). If T and S are independent variables (as with a standardized test that is invalid, failing to predict success), then the covariance between T and S is 0, and the correlation coefficient is 0.


$$\rho_{T,S}=\frac{COV(T,S)}{\sigma_{S}\sigma_{T}}$$ Eq 1

Eq 1 is expressed in terms of the full population under consideration (e.g., if all applications were admitted) but any attempt to measure the correlation is necessarily based on a subset of the population (a sample correlation, *r*, rather than the population correlation, ρ). A ’restricted range’ selection effect occurs when only considering a narrow range of values (e.g., an admissions policy that only admits applicants with the top 5% of test scores). In such a situation, the observed variance of the standardized test may be similar to the variance expected from test-retest reliability of the test (e.g., the variability among the top 5% of test scores might be comparable to the variability seen when one student takes the same test multiple times). Thus, there is no ’true’ variability in the latent characteristic(s) that the test hopes to measure when dealing with a restricted range; all admitted students are roughly the same in terms of the latent characteristics captured by the standardized test. In this case there cannot be any meaningful covariance with academic success even if the standardized test score is a valid predictor. As applied to predictive validity of standardized test scores, range restriction correction can be applied based on the variance of the standardized test score in the pool of applicants as compared to the variance of the standardized test score among admitted students [8]. However, we are unaware of any technique that can correct for a compensatory selection effect.

In the simulations presented below, even rejection of just 5% of the applicant pool can nonetheless create a compensatory selection effect that produces spurious correlations (e.g., turning an absent correlation with academic success for an invalid test into what appears to be a negative correlation with academic success). Furthermore, even after a highly selective admissions process, standardized test scores of admitted students can nonetheless show substantial variability, covering much of the range of possible values, and yet there is still a selection effect that eliminates the positive correlation with success that would otherwise exist if all applicants were admitted. Thus, *a data sample with sufficient variability offers no protection from a compensatory selection effect*. More to the point, variability in the standardized score is precisely the problem with a compensatory selection effect because *the selection process introduces negative covariance between the predictors* (a ’collider bias’ or ’Berkson’s paradox’ effect).

Consider a situation in which a highly selective school uses a holistic evaluation of a student’s application, using all parts of the application rather than merely using a specific cutoff based on standardized test scores. Indeed, the reason that school applications include multiple components is precisely because standardized test scores are thought to only measure some of the important attributes for academic success, while failing to measure less easily quantifiable attributes such as perseverance, diligence, and creativity, which are arguably just as important. With a holistic evaluation process, which is likely the norm for many admissions processes, the different components of the application may trade off against each other in a compensatory manner to determine who is admitted.

Because the elements of an application trade off against each other in a selective admissions process, this introduces a negative correlation between standardized test scores and the other predictors (e.g., an admitted student with a an unusually low test score is more likely to have exceptionally good reference letters). If the other components of the application are valid predictors of academic success, this negative correlation among the predictors works against finding any correlation between the standardized test and academic success. Thus, if the correlation between test scores and academic success would have been positive among the entire pool of applicants (i.e., the test is a valid predictor), compensatory selection can counteract this positive correlation and produce an absent correlation among the admitted students. Furthermore, if the correlation would have been absent among the entire pool of applicants–that is, if the standardized test is truly not predictive of academic success–compensatory selection will cause a negative correlation between the test scores and academic success among the admitted students.

Prior work has considered the role of compensatory selection in school admissions [9, 10], although these studies did not outline how these effects differ from restricted range, and did not consider how compensatory selection can distort results when applied to an invalid predictor. Here we address these issues with computer simulations.

## Simulation studies

To address the role of selection effects it is necessary to know what would have happened for rejected applicants if instead they had been accepted. However, it is impossible to know such counterfactuals with certainty. Instead, we use computer simulations to explore a range of possibilities. Each simulation makes mathematical assumptions regarding individual characteristics and the way these characteristics determine standardized scores and other predictors, as well as academic success. These assumptions are used to generate hypothetical applicants, and then an admissions process, with certain assumptions, is applied to select a subset of applicants. Because the characteristics of these hypothetical applicants are known, we can then examine the academic outcome for both accepted and rejected applicants.

We present a representative set of simulations chosen to highlight compensatory selection effects that were consistent across all simulations. In these simulations, academic success was defined as the binary outcome of degree completion. This representative set was drawn from a larger set of simulations that considered a wide variety of parameters and settings, including: 1) the number of latent characteristics underlying degree completion, e.g., compensation requires at least two latent characteristics and the reported simulations assumed four latent characteristics; 2) correlation patterns among the predictors as indicated by different factor-loading assumptions between predictors and latent characteristics; 3) different admissions committee policies, e.g., adding predictor values, multiplying predictor values, employing cut-off levels, etc.; 4) different levels of selectivity, e.g., accept top 5%, top 10%, top 30%; 5) different levels of measurement noise, i.e., test-retest reliability of predictors; and 6) different functional forms for the “true” relationship between latent characteristics and degree completion, e.g., completion probabilities are related to the addition or multiplication of latent characteristics, and different degrees of non-linearity between the scores and completion probability. The results were qualitatively similar across all variants, except where noted.

**Fig 1:**
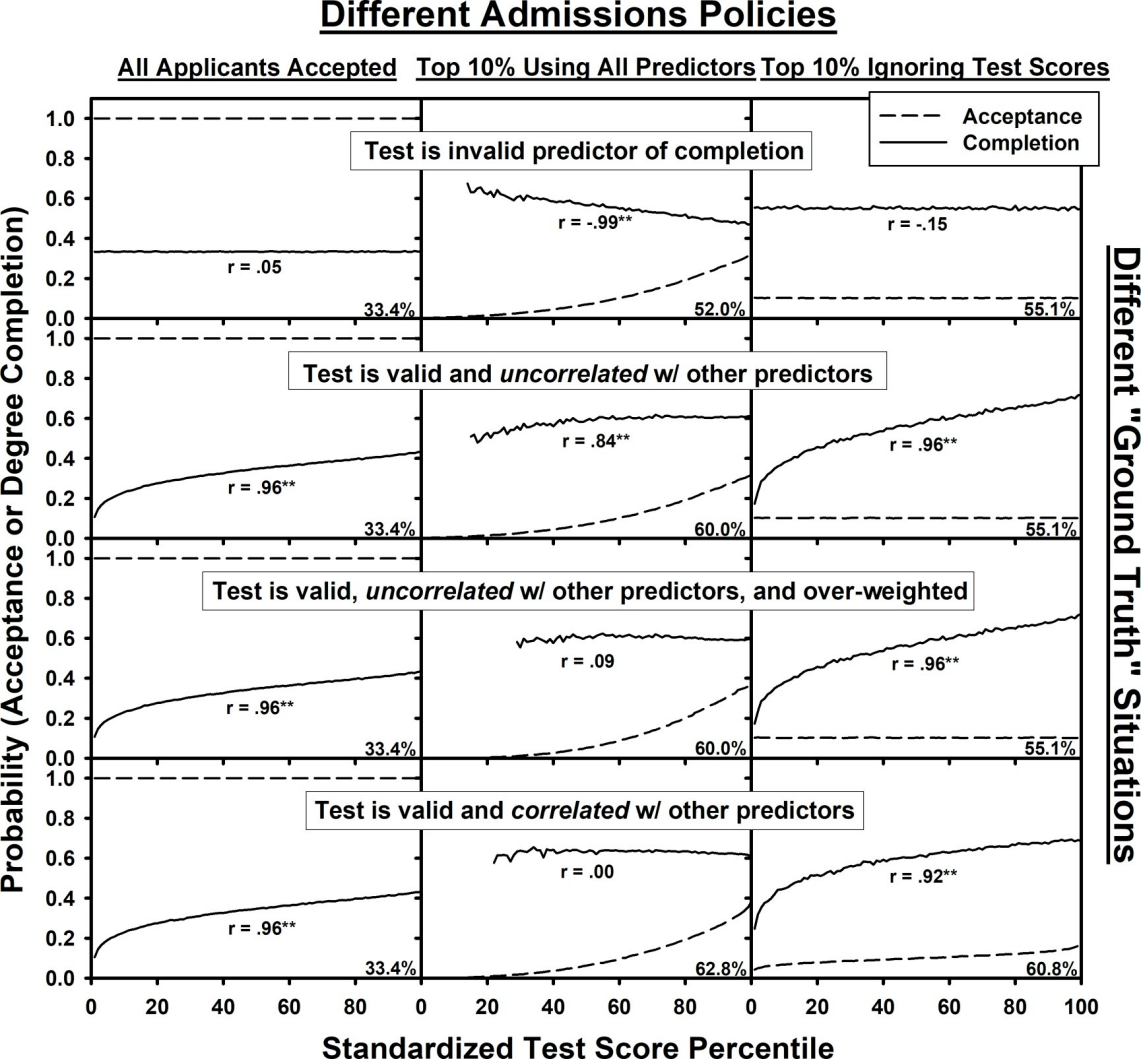
**Different ground truths.** The displayed simulation results compare three different admissions policies (columns) applied to four different “ground truth” situations (rows). Each graph plots both the probability of acceptance and the probability of degree completion for accepted students at each standardized test percentile. For the middle column, the admissions policy added up all predictors, including test scores, accepting the top 10% of applicants based on the summed score. The right column does the same, but without test scores. The percent numbers in the lower right-hand corner of each graph show the probability of degree completion averaged across all accepted students. For each simulation, the correlation (*r*-value) between test percentile and degree completion probability is reported and double stars (**) indicate significance values less than .001 (all other *r*-values had *p*-values greater than .05).

Distilling the most interesting and informative cases from these simulations, we report below four different “ground truth” situations (the four rows of Fig 1), capturing different possible states of the world for the applicant pool. The different ground truth situations correspond to different assumptions about whether the standardized test is a valid predictor of degree completion, whether the standardized test is correlated with the other predictors, and how much weight is placed on the standardized test during the admissions process. The four ground truth situations are: 1) the standardized test is not valid; 2) the standardized test is valid and uncorrelated with the other predictors; 3) the standardized test is valid and uncorrelated with the other predictors, but over-weighted; and 4) the standardized test is valid and correlated with the other predictors. For each ground truth, we report degree completion rates and correlations between degree completion and standardized test scores under three different admissions policies (the three columns of Fig 1): 1) accepting every student; 2) accepting the top 10% of applicants based on the summation of predictors, including standardized test score, i.e., a situation where a low standardized test score can be compensated for based on the other aspects of an application; and 3) accepting the top 10% based on the summation of predictors that did not include standardized test scores. We include the third policy to make predictions for schools that may decide to drop the use of standardized test scores in their admissions process.

All simulations in Fig 1 assumed four latent characteristics, i.e., psychological factors that cannot be directly observed, that were equally predictive of degree completion. The interpretation of these latent characteristics is completely arbitrary, and so they are given the labels of *Latent1*, *Latent2*, *Latent3*, and *Latent4*. Based on surveys of school admissions committees [11], applications were assumed to include four observed predictors that were equally important in determining a rank order of applications: **Test** scores, **Grades**, reference **Letters**, and a personal **Statement** (we adopt *italics* for latent characteristics and **bold** for observed predictors). For situations where the four predictors were assumed to be uncorrelated (see the top three rows of Fig 1), we assumed a one-to-one correspondence between each latent characteristic and one of the predictors. For the ground truth situation in which the **Test** is an invalid predictor, *Latent1* still determined degree completion, but in this case, we assumed that **Test** scores failed to measure *Latent1*. This was achieved by setting **Test** percentiles to random values drawn from a uniform distribution between 0% and 100%. Below we provide an explanation of how correlations between predictors were implemented.

### Simulation methods

The simulated applicant pool consisted of 10,000,000 individuals. Commented Matlab code can be found at: https://github.com/dhuber1968/CompensatorySelection.git

#### Latent characteristics and degree completion

For each individual, four latent characteristics were randomly sampled from a standard normal distribution. To make the simulation procedures more concrete in the descriptions below, consider a specific hypothetical applicant with latent characteristics of -0.158 for both *Latent1* and *Latent2* and 0.282 for both *Latent3* and *Latent4*. Degree completion was computed from these four latent characteristics via the following steps.

First, a "sufficiency probability" was calculated for each latent characteristic. This probability represents the likelihood that the applicant’s level on that single latent characteristic is sufficient to complete the degree. The sufficiency probabilities for each latent characteristic were calculated from a cumulative normal distribution with an underlying mean of -1 and standard deviation of 1. In this example, the hypothetical applicant would have a .8 sufficiency probability for *Latent1*, .8 sufficiency probability for *Latent2*, .9 sufficiency probability for *Latent3*, and .9 sufficiency probability for *Latent4*. A latent characteristic value of -0.158 for *Latent1* corresponds to a sufficiency probability of .8 because 80% of a normal distribution centered at -1 with a standard deviation of 1 falls below -0.158.

Second, the product of these four sufficiency probabilities determined the probability of degree completion (e.g., .8*.8*.9*.9 = .52). Thus, if an accepted applicant was fully sufficient on all four latent characteristics (1*1*1*1 = 1), degree completion was certain. If any of the four sufficiency probabilities was zero, failure was certain.

Finally, degree completion was determined from a Bernoulli coin flip based on this probability of PhD success. This procedure injects an element of randomness into degree completion, capturing the reality that factors unknown by admissions committees may underlie a student’s decision to drop out of a degree program. Continuing with the hypothetical applicant, there is a 52% chance that this individual will complete the degree if accepted. A slightly biased coin (one yielding heads 52% of the time) is flipped, and if heads, then this applicant succeeds, otherwise this applicant fails to obtain a degree.

#### Predictor variables and admissions policies

Values for each of the four predictors (**Test**, **Grades**, **Letters**, and **Statement**) were determined based on factor-loadings on the latent characteristics as follows. First, a linear combination of the latent characteristics was computed. The weights for the linear combination are discussed below. Second, standard normal measurement error was added for all four predictors (one full standard deviation, which corresponds to a fairly low test-retest reliability of .48 for the correlation between the percentile score when the same individuals take the standardized test twice). Finally, the resulting values were rank ordered across all individuals to determine a percentile score, which was then defined as the predictor value. This process was repeated for each predictor. The weights in the linear combination differed across factor-loading assumptions. To keep variance equal across the predictors, the sum of the squared factor weights was constrained to be 1. The factor-loading matrix for ground truth simulations with uncorrelated predictors (ground truths #2 and #3) was an identity matrix; that is, **Test** was based solely on *Latent1*, **Grades** was based solely on *Latent2*, **Letters** was based solely on *Latent3*, and **Statement** was based solely on *Latent4*. The identity matrix was also assumed for ground truth #1, except that **Test** scores were chosen randomly, rather than basing these scores on *Latent1*.

Table 1 shows the factor-loading matrix for ground-truth #4. These values are somewhat arbitrary and were chosen because they give rise to an absent correlation between test scores and degree completion, although many other choices for these factor loadings were also found to produce absent correlations. Other choices for the factor-loading matrix can result in positive correlations between **Test** scores and degree completion and even negative correlations. The simulation is an existence proof that correlated predictors *can* give rise to an absent correlation.

**Table 1:** Factor-loading matrix for ground truth #4.

	*Latent1*	*Latent2*	*Latent3*	*Latent4*
**Test**	1	0	0	0
**Grades**	.58	.71	.41	0
**Letters**	0	.41	.71	.58
**Statement**	0	0	0	1

Factor-loading weights applied to latent *characteristics* (columns) created observed **predictors** (rows).

Continuing the previous example for a single individual, the linear combinations for correlated predictors would be 1*-0.158 = -0.158 for **Test**, .58*-0.158 + .71*-0.158 + .41*0.282 = -0.088 for **Grades**, .41*-0.158 + .71*0.282 + .58*0.282 = 0.299 for **Letters**, and 1*0.282 = 0.282 for **Statement**. Standard normal noise was then added to these values. This process was repeated for all individuals resulting in 10,000,000 values for each predictor. Finally, the value of these predictors was transformed to percentile scores by rank ordering each predictor across all individuals.

Selective acceptance decisions were based on a weighted sum of the four predictors. The default weights were 1, with the following exceptions. When **Test** was over-weighted, the **Test** weight was 1.3. When the **Test** was not used in the admissions process, the **Test** weight was 0. Individuals with the top 10% weighted sums were accepted.

### Simulation results

#### Ground truth #1: The standardized test is an invalid predictor

The first row of Fig 1 implements the assumption that the **Test** is non-diagnostic, i.e., not predictive of degree completion. All latent characteristics and observed predictors for all simulated individuals for all three graphs of the top row were generated in same manner, with the only difference among the three graphs of the top row being the admissions policy applied to the hypothetical applicants. First, consider the upper left-hand graph in which the admissions policy accepts all applicants. There are no selection effects for this admissions policy and, correspondingly, there is no correlation between **Test** percentile and degree completion, accurately reflecting the assumed ground truth (the reported *r* value of .05 is not reliable and just reflects noise in the simulation–across runs of the simulation, the *r* value is just as likely to be slightly negative as it is slightly positive). The value in the lower right corner of the graph, 33.4%, provides the probability of degree completion averaged across accepted students (in this case, the entire population of applicants).

**Fig 2:**
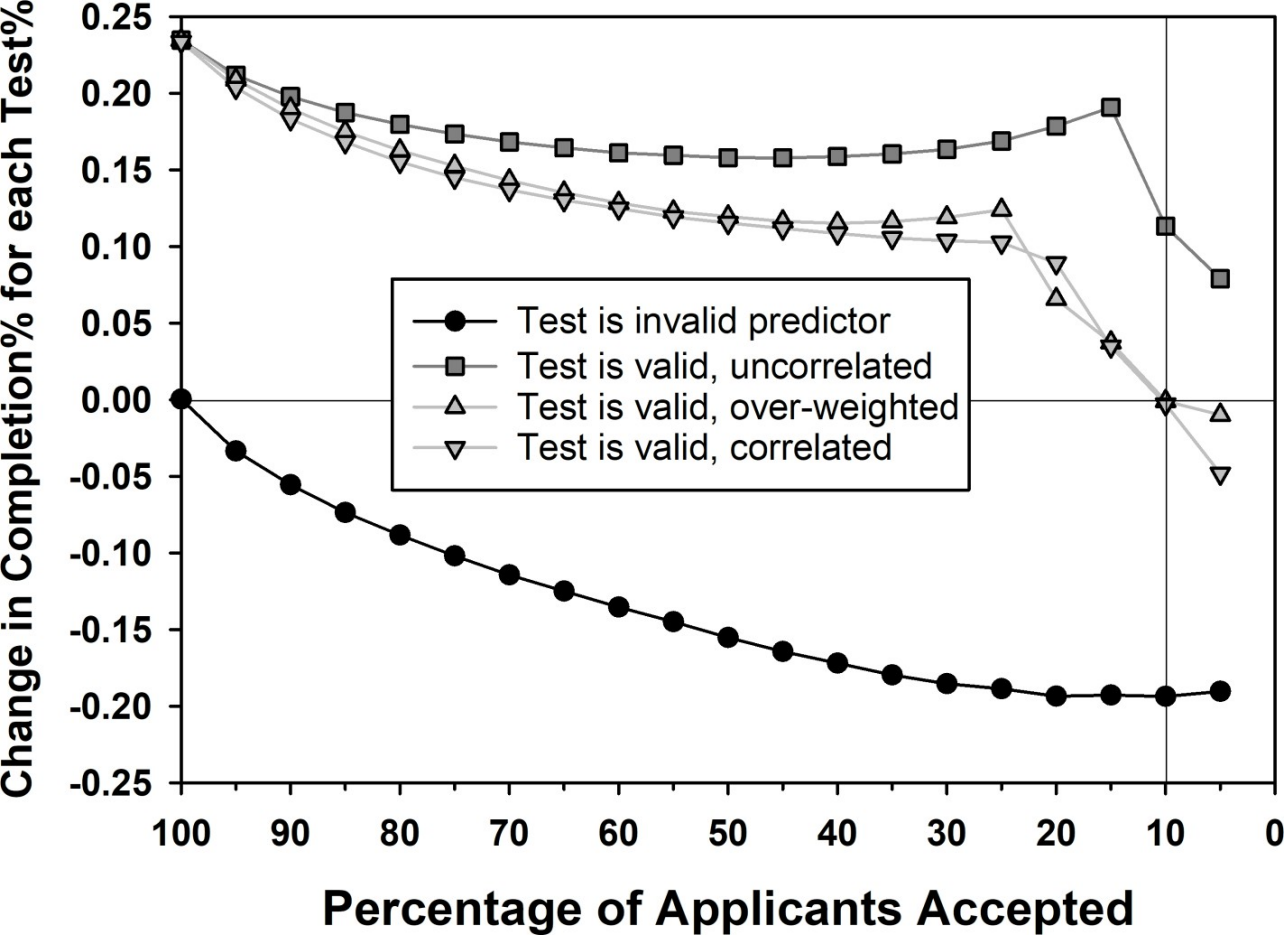
Test score regression slope as function of acceptance percentage. This graph shows the role of selective acceptance, ranging from 100% (accept everyone) to 5% (accept top 5%) in 5% steps for the same four ground truths shown in Fig 1. The graph shows the regression slope between **Test** scores and degree completion on the *y*-axis in units of percent completion change with each change of **Test** percentile. For instance, a value of .2 indicates that a change of 10% in terms of **Test** percentiles corresponds to a 2% increase in the probability of degree completion. The vertical line at 10% indicates the level of selectivity shown in Fig 1 and the results at this level are identical to the results in Fig 1.

Next, consider a selective admissions policy that accepts the top 10% of students based on a summed score across all four predictors (upper middle graph of Fig 1). Paradoxically, this results in a strong negative correlation between **Test** scores and degree completion. *More importantly, as reported below in the second set of simulations associated with Fig 2, this negative correlation occurs even if 95% of students are accepted (i.e., even if the selection process only rejects 5% of applications).* This negative correlation occurs because admissions committees are using **Test** scores in a compensatory manner: Students with lower **Test** scores may still gain acceptance if their other predictors indicate that they are a competitive applicant, and applicants with higher **Test** scores may be accepted despite weakness for other predictors. In the simulation captured in Fig 1, the selective admissions process introduced a negative correlation of *r* = -.248 among the accepted applicants between **Test** scores and **Grades**, **Letters**, and the personal **Statement**. Because these other predictors were valid predictors of degree completion, this compensatory selection created a negative correlation with degree completion (i.e., a student with a lower **Test** score is more likely to excel on these other three predictors and is therefore more likely to compete their degree). Because 3 of the 4 measures were valid, this admissions policy improved the chances of completion from 33.4% to 52.0%.

Finally, consider what happens if **Test** scores are dropped from the admissions process (upper right-hand graph). In this case, because the **Test** fails to reflect *Latent1* (it is non-diagnostic, merely adding random noise to the application), the effect of dropping it from the admissions process is to increase probability of degree completion to 55.1%. Furthermore, the true (lack of) correlation between **Test** scores and degree completion is revealed.

Across the large set of simulations discussed previously, all but two attempts to implement invalid **Test** scores revealed the same results as seen in the first row of Fig 1, with a negative correlation emerging between **Test** scores and degree completion, when selective acceptance is based on **Test** scores along with other predictors. We do not report the details of these two exceptions in detail because they are unlikely to occur in reality. The first of these two exceptions is a degenerate case in which none of the predictors are valid (in this case, none of the predictors correlate with degree completion regardless of the admissions policy). The other exception is an admissions policy that uses **Test** score as a strict cutoff but does not allow those making admissions decision to see the **Test** scores (they are only told that the applicant has exceeded the minimum). In this strange ground truth situation, instead of a negative correlation, there is no correlation between **Test** scores and degree completion. However, *if admissions committees can see* ***Test*** *scores*, *they are likely to use them in a compensatory manner* for applicants with above-minimum **Test** scores; that is, an applicant with perfect **Test** scores might be accepted despite other weaknesses, whereas an applicant just barely exceeding the **Test** cut-off would only be accepted if there were other strengths in their application. Thus, there would be a negative correlation between **Test** scores and degree completion owing to this compensation.

#### Ground truth #2: The test is valid and uncorrelated w/ other predictors

The second row of Fig 1 considers a situation in which all four of the predictors are equally predictive of degree completion and, furthermore, uncorrelated with each other. As seen in the left-hand graph, there is a strong correlation between **Test** scores and degree completion when all students are accepted. As seen in the middle graph for this ground truth, the inclusion of **Test** scores in the selection process tempers this positive correlation (the *R*^*2*^ percent variance accounted for drops from .92 drops to .71) and with a small sample, it might be hard to detect the weakened positive relationship between completion and **Test** scores. This use of all four predictors for a top 10% selection process increased degree completion from 33.4% to 60%. Furthermore, as seen in the right-hand graph, when **Test** scores are ignored in the admissions process, the average degree completion rate drops from 60% to 55.1%. This occurs because when **Test** scores are dropped from consideration, admissions committees no longer have accurate information about one of the latent characteristics necessary for degree completion.

#### Ground truth #3: The test is valid, uncorrelated, and over-weighted

The third and fourth rows of Fig 1 demonstrate two different ways in which selection effects can result in no correlation between **Test** scores and degree completion even though **Test** scores are predictive of degree completion when all applicants are accepted. The third row is the same simulation as the second row, except that the admissions policy assigns more weight to **Test** scores in producing an aggregate score for the top 10% selection process. In other words, even though the four predictors are equally diagnostic of degree completion, admissions committees over-weight **Test** scores. This eliminates accepted students with lower **Test** scores, as seen in the middle graph, resulting in little relationship between **Test** scores and degree completion (*r* = .09). Nevertheless, this over-weighting of **Test** scores is a subtle effect, and the completion probability is nearly unchanged from the second row (it differs only in the fourth decimal place). Furthermore, despite over-weighting **Test** scores, there is still a 4.9% drop in degree completion probability when **Test** scores are ignored, as seen in the right graph.

#### Ground truth #4: The test is valid and correlated w/ other predictors

In reality, the predictors are likely to be correlated. For instance, a student with good **Grades** likely has strong reference **Letters**. There are many possibilities for these correlations, with specific correlation matrices arising from specific factor-loading assumptions for determining observed predictors from latent characteristics.

If **Test** scores are invalid predictors (i.e., Ground Truth #1), it does not matter how these correlations are implemented, and all choices result in a negative correlation between **Test** scores and degree completion for a selective admissions process based in part on **Test** scores (except for a degenerate case where **Test** scores are perfectly correlated with all of the predictors, in which case all of the predictors are invalid). In contrast, for a ground truth of valid **Test** scores, some factor-loading choices result in a positive correlation between **Test** scores and degree completion among accepted students while others result in a negative correlation between **Test** scores and degree completion among accepted students, even though the underlying reality assumes that **Test** scores are predictive of completion in the applicant pool.

In the simulations reported in the bottom row of Fig 1, the chosen factor-loadings between latent characteristics and predictors resulted in a correlation of .28 between **Test** scores and **Grades**, a correlation of .28 between **Grades** and reference **Letters**, and a correlation of .28 between reference **Letters** and personal **Statements**. This situation produced an absent correlation (*r* = .003) between **Test** scores and degree completion as seen in the middle graph. This factor-loading was chosen as just one example that produces the lack of correlation, although this result is not unique, and other factor-loadings also fail to produce a correlation. *Critically*, *this occurred even though nearly 80% of the* ***Test*** *score range is included among the sample of accepted students*–although an applicant with a **Test** score of 20% was very unlikely to be accepted, some applicants with such low scores were accepted because the rest of their application was exceptional. *Thus*, *this elimination of the true positive correlation was not caused by a restricted range in the sample of* ***Test*** *scores*.

The point of this simulation is to consider a case in which the underlying attribute(s) measured by the **Test** may also be reflected in the other predictors. This is often the rationale for dropping standardized tests, i.e., that the information contained in a standardized test may be partially redundant with the other measures. But in fact, despite this partial redundancy, when **Test** scores were dropped from the admissions process (right graph), the simulation revealed that completion rates dropped from 62.8% to 60.8%, and the underlying positive correlation between **Test** scores and degree completion was revealed. If the assumption of a valid **Test** that is correlated with the other predictors (i.e., Ground Truth #4) is close to reality, these results suggest that there may be a modest cost to degree success when dropping a standardized test. Whether this modest cost outweighs the potential benefits is a difficult policy decision currently faced by school admissions offices.

#### Different levels of selectivity

The results reported in Fig 1 compare acceptance for all students versus only the top 10% of applicants. To assess the generality of these selection effects, we varied the acceptance percentage from 100% to 5% in 5% increments for each of the four ground truth situations under the assumption that **Test** scores are used in the admissions process. Fig 2 shows the linear regression slope between **Test** percentile and degree completion percentage. For instance, a slope of .2 means that for each increase of one **Test** percentile there is a corresponding increase in completion percentage of .2 (or, equivalently, an increase in completion by 20% across the entire range of **Test** scores).

As seen in the Fig 2, if the standardized test is invalid, yet used in the admissions process, *any level of selectivity results in a negative slope*, which becomes progressively more negative with increasing selectivity (solid circles in the lower half of the graph). To be clear, this indicates that *even if only a small fraction of the applicant pool is rejected*, *there will be a negative correlation between Test scores and degree success if the Test is an invalid predictor*. Thus, the failure to observe a negative correlation (i.e., an observation of a positive correlation or even a correlation that is reliably zero) will reject the hypothesis that the standardized test is an invalid predictor, unless all applicants were accepted. In contrast, if the standardized test is valid, yet uncorrelated with the other predictors, the regression slope is positive even for extreme levels of selectivity. The third and fourth ground truths are nearly identical, with a nearly constant level of positive regression slope up until 25% accepted, followed by a precipitous drop in the regression slope with further increases in selectivity, crossing the zero slope point for 10% acceptance, as highlighted with the vertical line (the simulations at 10% are identical to the ones shown in Fig 1). For a selectivity of 5%, the case where **Test** scores are valid and correlated with the other predictors results in a negative relationship between **Test** scores and degree completion.

The precipitous decreases in the slope starting at 15% for ground truth #2 and starting at 25% for ground truth’s #3 and #4 occur because of the non-linear relationship between the **Test** and degree completion (see the first column of Fig 1 for ground truths #2-#4). More specifically, these are the levels of selectivity at which applicants with very low **Test** scores are no longer accepted, which cuts off the non-linear rapid rise portion of the relationship between **Test** scores and degree completion. With the selected students only drawn from the linear slow rise portion of the relationship between **Test** scores and degree completion, compensation between predictors is apparent, and the slope values rapidly decrease with further increases in selectivity.

Across the entire range of acceptance percentages in Fig 2 and across all “ground truth” assumptions, an absent relationship was found only when the standardized test was invalid and everyone was accepted, or when the test was valid and yet either over-weighted or correlated with other predictors (for these particular parameter values, a slope of zero occurred at 10% acceptance, but this zero-crossing would likely occur at a different level of selectivity with different parameter values). This simulation highlights that our results are not specific to a single school with a 10% acceptance rate. Indeed, if students fail to gain acceptance to a highly selective school, they may ultimately accept enrollment at a less selective school (it is for this reason that the simulation results are reported in terms of ’accepted’ students, rather than ’admitted’ students, to acknowledge that students may turn down acceptance to go elsewhere). However, provided that some students fail to obtain acceptance at any school, even this very minimal censoring in the data sample will cause an invalid standardized test to produce a negative correlation with subsequent success (e.g., the 5% accepted result in Fig 2 for an invalid test). More specifically, the applicants that fail to gain acceptance to any school likely have weakness in all components of their application. Because they have been removed from the sample, all students in the sample either have strength in all components of their application or possess compensatory strengths such that their application is good enough for acceptance at a less selective school. Thus, because degree programs almost always reject some applicants, these results indicate that an observed lack of correlation between standardized test scores and degree completion provides evidence that the standardized test is predictive of success; *if the test were invalid*, *the correlation would be negative rather than absent*, *even if the school accepted 95% of applicants*.

## Discussion

To summarize, these simulations illustrate that a lack of correlation between standardized test scores and degree completion, following an admissions process in which predictors are compensatory, provides evidence that the test is an effective predictor of degree completion. If the test were invalid and yet used in a selective admissions process (even one that only rejected 5% of applicants), there would be a negative correlation between test scores and subsequent success in the degree program.

After performing the simulations presented above, we learned of an unpublished simulation study that considered a selective admissions process based on the use of two predictors [10], which is very similar to our own in some respects. Using slightly different mathematical assumptions, this study demonstrated that compensatory selection can reduce and even reverse the positive correlation between standardized test scores and academic success that would occur if all students were admitted. Similar to our simulations, this study considered what happens with different types of admissions policies (e.g., different levels of selectivity, use of cutoffs, over-weighting of standardized test scores, etc.). However, our study differs in two important ways. First is a simple terminological difference: We term the present phenomenon ’compensatory selection’ rather than ’restricted range,’ considering that these two selection effects are not actually identical (restricted range is a reduction in variance for the predictor whereas compensatory selection is the creation of negative covariance between the predictors owing to the selection process). Researchers often examine the variability of standardized test scores in their data sample to check whether their results might suffer from a restricted range problem. However, doing so offers no protection from a compensatory selection effect. Thus, it is critical to make clear the conceptual difference between these different selection effects. Second, our study differs by considering the hypothetical situation in which the standardized test is actually an invalid predictor. This hypothetical demonstrates that a compensatory selection effect can occur even when only a small percentage of applicants are rejected. In our simulations, even a 5% rejection rate created a negative correlation between standardized test scores and academic success if the standardized test offers no predictive power in either direction for the full pool of applicants. Thus, one of the central contributions of the present study is to show that unless all students are admitted, any failure to find a negative correlation between standardized test scores and subsequent success (e.g., a reliably positive correlation or a Bayes factor indicating strong support for a null correlation) indicates that the test has some predictive validity. Nevertheless, we strongly urge the interested reader to examine the simulations in [10], which complement our own.

The present simulations are an existence proof regarding a ’compensatory selection’ effect when evaluating standardized scores. However, there are real-world complexities that should be carefully considered, which provide important caveats to these conclusions. For instance, many studies examine data from a wide range of degree programs that each have different levels of selectivity, different levels of support, and different admissions procedures. In contrast, these simulations assumed that all degree programs adopt the same procedures and provide the same level of support. These differences between degree programs could potentially change the conclusions in a qualitative manner *if the results were analyzed without regard to such differences* (e.g., an analysis that collapsed the data across degree programs). Because some degree programs provide more financial support than others, students in well-supported programs may be more likely to complete their degrees (i.e., top-tier universities have larger endowments, allowing them to offer greater financial support and to provide better facilities and access to world renowned faculty). These top-tier programs are likely to be more selective, meaning that their students will have higher average standardized test scores. A correlation analysis that collapsed *across* schools and universities that differed in this regard could artificially produce a positive or absent correlation between test scores and academic success owing to this unacknowledged confounding factor even if the underlying truth was an invalid standardized test (i.e., even if the underlying correlation *within* programs was negative, owing to selection effects).

Most researchers are aware that differences between schools can be a confounding factor when assessing the validity of standardized scores, and they either consider degree programs separately [2], only collapse across degree programs that are highly similar [3], or include program as a factor in a multiple regression analysis to factor out such differences [1]. After controlling for differences between schools that might otherwise produce a spurious positive correlation with degree completion, these studies failed to find any relationship between standardized test scores and degree completion and concluded on this basis that the standardized test lacked predictive validity. However, our results indicate that their conclusion should have been exactly the opposite. If the test was an invalid predictor among the entire population of applicants, there should have been a negative correlation rather than an absent correlation because some applicants were likely rejected from all schools. Demonstrating the powerful nature of the compensatory selection effect, elimination of just 5% of the applicants from the sample would create a spurious negative correlation with degree completion for an invalid standardized test. Therefore, the failure to find such a negative correlation supports the predictive validity of the test.

As noted above, these simulation results hold over a range of implementation details and parameter settings, but two assumptions are critical: first, that the standardized test is not *perfectly* correlated with other measures; and second, that admissions committees use multiple predictors in a compensatory manner. Given these assumptions, if a standardized test is a valid predictor of success, then the observed relationship between test scores and academic success among admitted students can be slightly positive, negative, or absent, depending on the degree of selectivity and other factors. In contrast, if a standardized test does not predict success among the pool of applicants, then the correlation between the test scores and academic success among admitted students should be strongly negative even for moderate levels of selectivity. Thus, owing to a ’compensatory selection’ effect, the failure to find a negative correlation provides evidence that a standardized test is predictive of academic success.
